# Linking Empowering Leadership and Organizational Citizenship Behavior Toward Environment: The Role of Psychological Ownership and Future Time Perspective

**DOI:** 10.3389/fpsyg.2019.02612

**Published:** 2019-11-29

**Authors:** Meiqin Jiang, Huaying Wang, Mingze Li

**Affiliations:** ^1^College of Economics and Management, Huazhong Agricultural University, Wuhan, China; ^2^School of Foreign Languages, Wuchang Shouyi University, Wuhan, China; ^3^School of Management, Wuhan University of Technology, Wuhan, China

**Keywords:** organizational citizenship behavior toward the environment, empowering leadership, psychological ownership, future time perspective, moderated mediation model

## Abstract

Organizations are confronted with increasing social responsibility to contribute to environmental sustainability. Employee organizational citizenship behavior toward the environment (OCBE) is considered essential to organizational environment performance. Drawing upon the theory of psychological ownership, the study investigated the effects of empowering leadership on employee OCBE by a sample of 374 employees in China. With the use of the bootstrapping technique in SPSS 25 to test our proposed moderated mediation model, results demonstrated a positive relationship between empowering leadership and OCBE through the mechanism of employee psychological ownership. Further, we found that the indirect effect is stronger when employees hold high rather than low future time perspectives. The theoretical implications for sustainability literature and practical implications for organizations striving for environmental sustainability are discussed.

## Introduction

With the glowing social concern about the environmental consequence of corporations, environmental sustainability is an inevitable consideration and a social responsibility in organizational settings (Starik and Marcus, 2000; Aguinis and Glavas, 2012; Kim et al., 2017). Internally, organizations are always concerned about the various negative effects of economic crisis on work conditions and employees (Giorgi et al., 2015; Mucci et al., 2016). Moreover, by engaging in pro-environmental behaviors, organizations can benefit from employees, customers, and overall performance in the long run (Maignan et al., 1999). Thus, because of external and internal concerns and the benefits of pro-environmental behaviors, organizations paid much attention to the development of environmental policy, and green human resource management practices (Smith and O’Sullivan, 2012), in which the top-down strategic initiatives for sustainability have been addressed.

However, the significance of the bottom-up influence initiated by individual pro-environmental conducts has been overlooked (Haugh and Talwar, 2010; Jenkin et al., 2011b; Boiral and Paille, 2012; Galpin and Lee Whittington, 2012). Understanding the role of individual employees in pro-environmental participation is important. On the one hand, only when individual employees actively respond to and support the strategic initiatives in the organizational level can the environment policy and advocacy be implemented (Daily et al., 2009; Jenkin et al., 2011a). On the other hand, individual employees may initiate and champion environmental sustainability in work situations (Paillé et al., 2013), and their contribution is vital to an organization’s environmental sustainability (Dilchert and Ones, 2012; Lulfs and Hahn, 2013). Therefore, managers care about how to stimulate individual employees’ participation in environmental sustainability.

Sustainability behaviors are essentially non-mandatory work-related tasks but rather voluntary involvement. Daily et al. (2009) conceptualized the voluntary employee green behavior as organizational citizenship behavior toward the environment (OCBE). OCBE refers to “discretionary acts by employees within the organization not rewarded or required that are directed toward environmental improvement” (Daily et al., 2009, p. 246). Employees’ OCBE is a necessary supplement to the environmental sustainability system in the organization and is found to have a significantly positive relationship with organizational environmental performance (β = 0.31; *p* < 0.01) (Paillé et al., 2013).

Why do employees engage in OCBEs? Some scholars have examined the factors that promote employee’s OCBE at work. In the organizational level, Daily et al. (2009) proposed that corporate social performance positively influenced individual’s OCBEs. Empirically, Paillé et al. (2013) found that strategic human resource management can predict employees’ OCBEs. Research also indicated that if organizations hold a positive attitude toward the environment, followers are more likely to engage in OCBEs (Erdogan et al., 2015; Lamm et al., 2015; Tuan, 2017). In terms of factor in individual levels, scholars have mainly investigated the influence of employee organizational commitment (Daily et al., 2009; Mesmer-Magnus et al., 2012; Paille et al., 2013; Erdogan et al., 2015; Temminck et al., 2015; Stritch and Christensen, 2016), and employee environmental concern (Daily et al., 2009; Temminck et al., 2015). Furthermore, given leaders play a critical role in motivating and shaping employee behaviors (LePine et al., 2016; Qian et al., 2018), an emerging attention focused on the role of leaders’ support behavior in employee pro-environmental behaviors (Smith and O’Sullivan, 2012; Paillé and Boiral, 2013; Lamm et al., 2015; Saifulina and Carballo-Penela, 2017; Wesselink et al., 2017; Priyankara et al., 2018).

Unfortunately, noticeably missing from research attention is how leadership style impacts employees’ OCBEs. Leadership style serves critical roles in achieving goals in work situations (McColl-Kennedy and Anderson, 2002; Turner and Müller, 2005), among which empowering leadership has assumed special importance (Srivastava et al., 2006), as it is consistent with the trend toward offering greater authority and self-responsibility to employees for better coping with a changing external environment (Takeuchi et al., 2005). Leaders sharing powers with followers can enhance employees’ motivation and investment in work (Griffin et al., 2007; Martin et al., 2013). To the extent that OCBEs are discretional involvement in work, a central question to ask is whether empowering leadership is associated with employees’ OCBEs. The extant researches provide no answers to this question.

**FIGURE 1:**
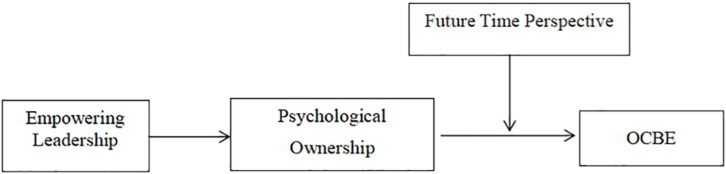
The Moderated Mediation Model.

To address this question, the present study investigates the relationship between empowering leadership and employee OCBEs. To link empowering leadership and employee OCBEs, we further draw on the theory of psychological ownership (Pierce et al., 2001, 2003; Van Dyne and Pierce, 2004) to clarify the effect of empowering leadership on followers’ OCBEs. Psychological ownership in work situations refers to feelings of possession toward the organization (Van Dyne and Pierce, 2004). We propose that empowering leadership might engender employee psychological ownership by offering participation, autonomy, responsibility, and expression of confidence in followers (Ahearne et al., 2005; Zhang and Bartol, 2010), which in turn motivates employee OCBEs. Finally, we also wonder when the impact of empowering leadership on employee OCBEs will be stronger. We expect that employees with a strong future time perspective will respond more positively to empowering leadership and subsequent psychological ownership than will those with a weak future time perspective. The research model is depicted in Figure 1.

Taken together, the present study aims to make four insightful contributions. First, we contribute to the employee pro-environmental behavior literature by identifying empowering leadership as an impactful force for motivating employee OCBEs from a leader style approach. Extant research concerning leaders’ role in employee OCBEs has largely focused on how employee-perceived manager support behaviors or manager support toward the environment affect employee OCBEs (Smith and O’Sullivan, 2012; Paillé and Boiral, 2013; Lamm et al., 2015; Saifulina and Carballo-Penela, 2017; Wesselink et al., 2017; Priyankara et al., 2018). Little is known about how leadership style, especially empowering leadership, may serve as a motivator of employee discretional green behaviors. Second, the present study draws on the psychological ownership theory (Pierce et al., 2001, 2003; Van Dyne and Pierce, 2004) to clarify why empowering leadership will elevate employee OCBEs, offering a different theoretical lens to explain employee OCBEs, supplemented to perspectives from social exchange theory (Paillé and Mejía-Morelos, 2014; Temminck et al., 2015; Priyankara et al., 2018), planned behavior theory (Greaves et al., 2013), and self-determination theory (Graves et al., 2013). Third, by investigating the synergistic effect of individual value (i.e., future time perspective) in strengthening the positive influence of empowering leadership and psychological ownership on individual OCBEs, we link the empowering leadership, psychological ownership, individual value of time perspective, and employee pro-environmental behavior literature that has developed separately and consider the individual–contextual interactionist factor for employee OCBEs. Finally, the present study also contributes to the empowering leadership literature by examining whether employees are more inclined to conduct discretionary green behaviors (i.e., OCBEs) when empowered by leaders, suggesting the environmental implication of empowering leadership.

## Theoretical Background and Hypotheses

### Empowering Leadership and OCBE

Organizational citizenship behavior toward the environment is defined as “individual and discretionary social behaviors that are not explicitly recognized by the formal reward system and that contribute to a more effective environmental management by organizations” (Boiral, 2009, p. 223), which is related to, but distinct from, organizational citizenship behavior (OCB) (Organ, 1998; LePine et al., 2002). Lamm et al. (2013) illustrated the characteristics of OCBE to distinguish it from other related constructs, such as daily sustainability behaviors, employee green behavior, and OCB. First, OCBE occurs in the context of the workplace. Second, OCBE is voluntary and proactive behaviors initiated by employees. Third, the purpose of OCBE is to contribute to environmental sustainability and indirectly benefit from the environment performance and sustainability of the organization. Boiral and Paille (2012) identified OCBE as a three-dimensional construct, that is, eco-civic engagement, eco-initiatives, and eco-helping. Eco-civic engagement reflects voluntary participation in environmental events and programs organized by the organization. Eco-initiatives involve discretionary environmental behaviors in organizations, such as reducing pollution, saving resource and energy, and suggesting environment improvements. Eco-helping involves employees voluntarily helping colleagues to adopt environmental consciousness in the workplace.

Empowering leadership is conceptualized as “a practice, or set of practices involving the delegation of responsibility down the hierarchy so as to give employees increased decision-making authority in respect to the execution of their primary work tasks” (Leach et al., 2003, p. 28). Ahearne et al. (2005) conceptualized empowering leadership as leaders highlighting the meaningfulness of work, fostering participation in decision making, conveying confidence in high performance, and providing autonomy from bureaucratic constraints. Empowering leadership in this study is taken as a dyadic relationship between the supervisor and a focal employee in line with some past empirical studies (Robert et al., 2000; Ahearne et al., 2005; Zhang and Bartol, 2010).

Given the power-sharing nature of empowering leadership to enhance followers’ motivation and involvement at work, there are reasons to expect a positive relationship between empowering leadership and employee OCBEs. Form the access perspective, empowering leadership offers employees with a certain degree of participation in decision making, and empowered employees are granted more autonomy at work. In such a work situation, employees have a chance to take eco-initiatives, such as making suggestions to improve the environment (i.e., OCBEs). Moreover, they have more opportunities to arrange their resource and work schedule to engage in discretionary pro-environmental behaviors. Also, they can to influence colleagues to be environmentally friendly. From the motivation perspective, empowering leadership provides employees with power and autonomy, and empowered employees are more likely to conduct discretionary pro-organizational behavior to reciprocate leaders’ power sharing (Blau, 1964; Cropanzano and Mitchell, 2005). OCBEs are a kind of discretionary behavior that indirectly benefits an organization’s environment performance and sustainability, so empowered employees might put effort to perform such discretionary pro-environmental behaviors valued by the organization and voluntary participation in environmental events and programs organized by the organization. From the cognition perspective, empowering leadership helps employees better understand the meaningfulness of their work and expresses confidence in their ability to achieve high performance. Thus, employees are aware of their goals and what the organization values and have confidence in their ability to perform well at work. As a result, empowered employees have more available cognition and efficacy to initiate pro-environmental behaviors that will make a difference for the sustainability of the organization. In contrast, those who are not empowered by leaders have less access, motivation, and available cognition to perform OCBEs, which are not required by their work description. Accordingly, we propose the following hypothesis.

Hypothesis 1: Empowering leadership is positively associated with OCBEs.

### The Mediating Role of Psychological Ownership

Psychological ownership is defined as “the state in which individuals feel as though the target of ownership or a piece of that target is “theirs” (i.e., “It is mine!”)” (Pierce et al., 2001, 2003). According to psychological ownership theory (Pierce et al., 2001, 2003), individuals can feel psychological ownership by three routes: controlling the target, having greater knowledge of or being more familiar with the target, and investing themselves in a target (Pierce et al., 2001). As mentioned above, empowering leadership exerts influence on employees as leaders highlighted the meaningfulness of work, fostered participation in decision making, conveyed confidence in high performance, and provided autonomy from bureaucratic constraints (Ahearne et al., 2005). We suggest that these leadership behaviors are highly associated with followers’ psychological ownership.

Empowering leadership might promote psychological ownership in followers through four mechanisms (Ahearne et al., 2005). First, empowering leadership highlights the meaningfulness of followers’ work; therefore, employees have a better understanding of their goals and contributions, which will lead to an increased sense of familiarity with their work and organization. Thus, a sense of familiarity and knowledge of the organization are related to the development of employee psychological ownership. Second, empowering leadership encourages followers to participate in decision making, through which employees have greater control over their work conditions (Zhang and Bartol, 2010). Thus, when employees involve themselves into the development of their organization and control their work situation, they will form a sense of psychological ownership. Third, empowering leadership expresses confidence in followers’ ability to achieve high performance. Researches showed that such empowering behavior is positively related to employees’ self-efficacy (Ahearne et al., 2005; Kim and Beehr, 2017). Employees’ psychological ownership emerges when they have more confidence in and control of their work and organization (Pierce et al., 2003). Finally, empowering leaders provide followers a great degree of work autonomy. Work autonomy indicates that employees can control their work, which in turn increases their experience of psychological ownership (Pierce et al., 2001; O’driscoll et al., 2006; Mayhew et al., 2007). Thus, for all the above reasons, it is quite likely that empowering leadership will increase employees’ psychological ownership.

According to the psychological ownership theory (Pierce et al., 2001, 2003; Van Dyne and Pierce, 2004), when employees feel psychological ownership toward their organization, they become more attached to, protective of, and responsible for it. Specifically, building on Pierce et al. (2001, 2003) work, Avey et al. (2009) conceptualized psychological ownership as a four-dimensional construct: self-efficacy, belongingness, self-identity, and accountability. Therefore, if employees consider the organization as “theirs” (i.e., psychological ownership), they consider the organizational identity as a great part of the self, feel like owners in the organization, feel responsible for the sustainability of the organization, and believe they can successful achieve it. The attachment and pro-organizational motivation behind psychological ownership drive employees to protect and enhance sustainability (Van Dyne and Pierce, 2004). Researches have also indicated that psychological ownership is positively related to OCB (Avey et al., 2009; Wang et al., 2019) or other extra-role behaviors (O’driscoll et al., 2006). Thus, we suggest that employees with organizational psychological ownership will care about the sustainability of the organization and take more initiatives in citizenship behavior toward the environment in support of the organization.

In conclusion, empowering leadership highlights the meaningfulness of work, encourages employees to participate in decision making, expresses confidence in employees’ ability to achieve high performance, and provides employees with a great degree of autonomy (Ahearne et al., 2005; Zhang and Bartol, 2010). These empowering behaviors lead employees to feel psychological ownership toward the organization. Employees with a feeling of psychological ownership tend to be more responsible for the sustainability of the organization and display more OCBEs. Therefore, we propose the following hypothesis.

Hypothesis 2: Psychological ownership mediates the relationship between empowering leadership and OCBEs.

### The Moderating Role of Future Time Perspective

Organizational citizenship behavior toward the environments are influenced by individual attitudes (Daily et al., 2009; Norton et al., 2015). Norton et al. (2015) suggested that attitude can play a moderating role in the relationship between factors and employee green behaviors. For instance, Bissing-Olson et al. (2013) found that the positive relationship between positive affect and employee pro-environmental behavior is moderated by employees’ pro-environmental attitude; when employees hold a negative pro-environmental attitude, positive affect is more strongly related to employee pro-environmental behavior.

We suggest that employee future time perspective may strengthen the positive relationship between psychological ownership and OCBEs. Future time perspective refers to the extent to which an individual values future-oriented events (Strathman et al., 1994), which is conceptualized as a relatively stable interindividual difference in cognitive orientations (Strathman et al., 1994; Zimbardo and Boyd, 1999), and differs from the notion of future orientation raised by Hofstede (2003) that refers to a culture value. Future time perspective contains three major cognitive dimensions that involve the concern for the future, that is, future orientation, continuity, and affectivity (Kooij et al., 2018). High future orientation means an individual focuses on future events (Gjesme, 1979). High continuity indicates that an individual believes that their present action can influence future outcomes (Husman and Lens, 1999). High affectivity refers to attaching greater value to goals that can be reached in the future (de Volder and Lens, 1982), also known as “delay of gratification” (Mischel, 1961).

For employees who hold high future time perspective, they might conduct more OCBEs when feeling psychological ownership toward the organization. When employees feel possession of the organization (i.e., psychological ownership), they feel responsible for the well-being and sustainability of the organization. Those who value high future time perspective will focus on future events, consider the long-tern consequences of their extent behaviors, and place greater importance on the distant goals (Kooij et al., 2018). Therefore, they care about the future and long-term outcomes of the organization. OCBE is an extra role initiated by individuals to benefit the organization’s environmental performance (Boiral, 2009). We argue that psychological ownership drives employees to act pro-organizationally, while future time perspective directs employees to work pro-environmentally. In conclusion, when employees who value future time perspective feel psychological ownership toward the organization, they care about the sustainability of the organization, and thus are willing to initiate pro-environmental behaviors. In contrast, those with low future time perspective value present rather than future performance. Even when they feel psychological ownership toward the organization, their behaviors are less likely directed toward the environment and future goals than employees with high future perspective. Therefore, we propose the following hypothesis.

Hypothesis 3: Future time perspective moderates the relationships between psychological ownership and OCBEs such that the relationship will be stronger for those high in future time perspective.

### The Integrated Moderated Mediation Model

Taken together, the above considerations described a model in which empowering leadership is positively related to employee OCBE (Hypothesis 1) and where employees’ psychological ownership plays a mediating role in such a positive link (Hypothesis 2). However, the strength of the relationship between employees’ psychological ownership and OCBEs is suggested to depend on their future time perspective (Hypothesis 3). In sum, these hypotheses specify a moderated mediation model (Preacher et al., 2007), in which empowering leadership is positively and indirectly related to employee OCBEs, through employees’ psychological ownership, with this indirect linkage depending on the level of employee future time perspective (see Figure 1). As we predict strong (weak) linkages between employees’ psychological ownership and OCBE when they have high (low) future time perspective, we expect the following:

Hypothesis 4: Employee future time perspective moderates the positive and indirect relationship between empowering leadership and employee OCBE through employee psychological ownership. Specifically, the indirect positive effect through employee psychological ownership will be stronger when employee has high rather than low future time perspective.

## Materials and Methods

### Sample and Procedures

We collected the data by using an online method. The participants of this study were employees from various organizations in China. The survey was displayed by email at two time points separated by 1 month to reduce the potential for common source and common method biases (Podsakoff et al., 2003). At time 1, through sending a questionnaire link to the employees directly, their demographic information, environment value, empowering leadership, and psychological ownership were obtained from 406 participants. At time 2, employees were asked to report their OCBEs and future time perspective. All participants were informed that the study was for academic research alone and assured anonymity to increase respondent candidness. In return for their participation, participants who completed the whole survey received an incentive of around five dollars. Participants are aware that if they quit after filling out the first questionnaire or if they do not finish the questionnaires carefully, they cannot get the incentive. Researchers gave out cash-filled red envelopes through mobile payment to participants after verifying these questionnaires within 1 day.

The data-collecting process meets the standard of ethics. Before starting the data collection, this study consulted the Ethics Committee of Wuhan University of Technology, and the committee approved this research. According to the research design, the study did not violate any legal regulations or common ethical guidelines. Because the required participants were recruited online, the researchers did not distribute a written informed consent, and the consent of participants was obtained by virtue of survey completion.

Finally, we obtained 398 valid questionnaires at time 1 with a response rate of 98.03%, and a total of 374 employees were retained for data analysis at time 2 with a response rate of 93.97% (the total response rate is 92.12%). Supplemental analyses revealed that there are no significant differences in demographics or responses between the dropped questionnaires and retained ones. The sample included 198 (53%) men and 176 (47%) women. Participants indicated their age in following categories: below 18 (0.80%), 18–25 (23.00%), 26–30 (55.60%), 31–40 (14.40%), 41–50 (5.90%), 51–60 (0.30%), and above 60 (0). The average organizational tenure was 3.27 years (*SD* = 3.84). With regard to education level, 79.95% participants had completed a bachelor’s degree or higher.

### Measures

All survey items were measured on 7-point Likert scales ranging from 1 (strongly disagree) to 7 (strongly agree). The items were originally written in English and then translated into Chinese and then back-translated into English by two independent bilingual individuals to ensure equivalency of meaning (Brislin, 1980).

#### Empowering Leadership

We measured empowering leadership with a 12-item scale developed by Ahearne et al. (2005). This measure is of good quality; for instance, the reliability of the scale in the original paper was 0.88 (Ahearne et al., 2005), and the reliability was 0.89 in a recently published paper (Gao and Jiang, 2019). Employees indicate the extent to which direct supervisors engaged in enhancing the meaningfulness of work, fostering participation in decision making, expressing confidence in high performance, and providing autonomy from bureaucratic constraints. The sample items are “My manager helps me understand how my objectives and goals relate to that of the company” and “My manager makes many decisions together with me.” The coefficient alpha was 0.96 in our study.

#### Psychological Ownership

We measured psychological ownership by applying Van Dyne and Pierce’s (2004) measure. The original scale has seven items, four items from which are used to measure the psychological ownership in the Chinese context by dropping one item that referred to a mutual sense of ownership and two items that are difficult to translate into Chinese (Bernhard and O’Driscoll, 2011). The four-item scale is also used in the Chinese context in Peng and Pierce’s (2015) research; the coefficient alpha was 0.85 in their research, which suggested a good quality of the scale. The scale opens with the following statement: “Think about the home that you own or co-own with something, and the experiences and feelings associated with the statement “THIS IS MY HOUSE!” The following questions deal with the “sense of ownership” that you feel for the organization that you work for. Indicate the degree to which you personally agree or disagree with the following statements.” The sample items are “I sense that this organization is our company” and “I feel a very high degree of personal ownership for this organization.” The coefficient alpha was 0.91 in our study.

#### Future Time Perspective

Future time perspective was assessed using a 13-item future time perspective scale from the Zimbardo Time Perspective Inventory (Zimbardo and Boyd, 1999); this scale is of good quality and has been applied to many studies. For example, the reliability of the scale in Qian et al.’s (2015) work was 0.97. Respondents are asked to rate “How characteristic or true is this of you?” with 13 questions. Sample items are “I believe that getting together with one’s friends to party is one of life’s important pleasures” and “When I want to achieve something, I set goals and consider specific means for reaching those goals.” The coefficient alpha was 0.92 in our study.

#### OCBE

We used a 10-item scale developed by Boiral and Paille (2012) to measure OCBE. This scale is of good quality as the reliability of the scale was 0.94 in their subsequent study (Paille et al., 2013). Sample items are “I weigh the consequences of my actions before doing something that could affect the environment” and “I actively participate in environmental events organized in and/or by my company.” The coefficient alpha for OCBE was 0.94.

#### Control Variables

As prior researches have shown that demographic characteristics may influence the extent to which individuals engage in OCBE (Lamm et al., 2015), we controlled for the effects of demographic characteristics. We controlled for the effects of gender, age, education level, and organizational tenure in our analysis. Organizational tenure was measured by years. Gender, age, and education level were coded as dummy variables. For gender, 1 = “male” and 2 = “female”; for age, 1 = “below 18,” 2 = “18–25,” 3 = “26–30,” 4 = “31–40,” 5 = “41–50,” 6 = “51–60,” and 7 = “above 60”; for education, 1 = “high school and below,” 2 = “college degree,” 3 = “bachelor’s degree,” 4 = “master’s degree,” and 5 = “PhD degree.”

Furthermore, as individuals’ environmental concern is considered as an important factor that contributes to their behaviors’ target environment (Daily et al., 2009; Temminck et al., 2015), we controlled employees’ *environment value* by using a four-item scale developed by Fukukawa et al. (2007) to assess the social and environmental accountability of corporations and executives. Sample items are “Business executives should be held accountable for the effects of their decisions on the environment” and “Corporations should be held accountable on issues relating to environmental responsibility (e.g., emissions, effluents and waste; energy usage; effects on biodiversity).” This scale is widely used in many studies and provided good quality. The coefficient alpha was 0.93 in our study. We also controlled the alternative explanation by *organizational commitment*, which has been shown to have a positive effect on employee OCBEs (Daily et al., 2009; Mesmer-Magnus et al., 2012; Paille et al., 2013; Erdogan et al., 2015; Temminck et al., 2015; Stritch and Christensen, 2016). Organizational commitment was measured by an eight-item affective commitment scale developed by Allen and Meyer (1990). Sample items are “I would be very happy to spend the rest of my career with this organization” and “This organization has a great deal of personal meaning for me.” This scale is widely used in many studies and provided good quality (e.g., McCormick and Donohue, 2019; Tan et al., 2019). The coefficient alpha was 0.94 in the present study.

### Analysis Strategies

This study first performed a confirmatory factorial analysis (CFA) to examine the fit of the measurement model by AMOS 22, and the model fit was assessed by some goodness-of- fit indices, such as comparative fit index (CFI) and standardized root mean square residual (SRMR). Second, we performed statistical analyses to assess the severity of common method bias of the present data. Third, we conducted a correlation analysis using SPSS 25.

Then, study hypotheses were assessed by conducting hierarchical regression analysis and bootstrapping indirect or moderated indirect effect analysis in SPSS 25. To test Hypothesis 1, we regressed OCBE on the control variables and on empowering leadership. To address Hypothesis 2, we regressed psychological ownership on empowering leadership and then regressed OCBE on control variables, empowering leadership, and psychological ownership. We also performed bootstrapping procedures to assess the indirect effect between empowering leadership and OCBE through psychological ownership. To examine Hypothesis 3, the interaction term of psychological ownership and future time perspective was included in the regression model of OCBE. Before being added to the regression, all the independent variables, except dummy variables for age, gender, and education, were grand-mean-centered to reduce multicollinearity problems (Aiken et al., 1991). To test the moderated mediation relationship in Hypothesis 4, we followed the steps provided by Preacher et al. (2007). This approach allows formal significance tests of the indirect relationship between empowering leadership and OCBE, as transmitted by psychological ownership, at the mean value of future time perspective and at one standard deviation below and above the mean, which can be achieved using the Process Procedure for SPSS written by Hayes (2013) and bootstrapping procedures (Edwards and Lambert, 2007; Preacher et al., 2007).

## Results

### Confirmatory Factor Analyses

Prior to testing the hypothesized relationships, we conducted CFA using AMOS 22 to assess the quality of our survey measures. The results of the CFA are presented in Table 1. As shown in Table 1, the hypothesized four-factor measurement model (empowering leadership, psychological ownership, future time perspective, and OCBE) provided good fit to the data (χ^2^/*df* = 3.24, *p* < 0.001; CFI = 0.92; TLI = 0.91; SRMR = 0.04; RMSEA = 0.08), better than all alternative three-factor, two-factor, and one-factor models (see models in Table 1). The factor loadings ranged from 0.79 to 0.87 for empowering leadership, from 0.91 to 0.97 for psychological ownership, from 0.83 to 0.90 for future time perspective, and from 0.89 to 0.95 for OCBE. All items for these four focal variables displayed adequate loadings. These findings demonstrate convergent validity and discriminant validity of the measures of our focal constructs.

**TABLE 1:** Confirmatory factor analysis results.

**Model**	**χ^2^**	***df***	**χ^2^/*df***	**CFI**	**TLI**	**SRMR**	**RMSEA**	**CI 90% RMSEA**
Four-factor model	2,252.46	696	3.24	0.92	0.91	0.04	0.08	(0.074,0.081)
EMP + PO, FTP, OCBE	3,857.36	699	5.52	0.83	0.82	0.07	0.11	(0.107,0.113)
EMP, PO + FTP, OCBE	7,643.58	699	10.94	0.63	0.61	0.27	0.16	(0.160,0.166)
EMP, PO, FTP + OCBE	7,306.39	699	10.45	0.65	0.63	0.18	0.16	(0.156,0.162)
EMP + PO + FTP, OCBE	9,070.59	701	12.94	0.56	0.53	0.25	0.18	(0.175,0.182)
One-factor model	12,773.09	702	18.20	0.36	0.32	0.28	0.21	(0.211,0.218)

**N* = 374. EMP, empowering leadership; PO, psychological ownership; FTP, future time perspective; OCBE, organizational citizenship behavior toward the environment; CFI, (Bentler’s) comparative fit index; TLI, Tucker-Lewis index; SRMR, standardized root mean square residual; RMSEA, root mean square error of approximation; CI, confidence interval.*

### Common Method Bias

As variables are all self-reported, there exists a potential problem for common method bias.

Firstly, we adopted procedure control methods recommended by researchers (Podsakoff et al., 2003, 2012) to reduce common method bias. For example, we used a time-lag design to collect data; that is, employees rated the dependent variables and moderating variables 1 month after independent variables to enhance the nature of causality in our study, and we also took effort to allow respondent anonymity and reduce evaluation apprehension. Further, the interaction effect is found to be weakened rather than strengthened by common method bias (Siemsen et al., 2010; Podsakoff et al., 2012); thus, a significant interaction effect can indicate that common method bias did not present a bias.

Then, we performed statistical analyses to assess the severity of common method bias. We conducted Harman’s single-factor test in SPSS 25, and results showed that four factors were present (i.e., empowering leadership, psychological ownership, future time perspective, and OCBE), and the most covariance explained by one factor was 38.54%, suggesting that common method bias was not a serious contaminant in our study (Podsakoff et al., 2003). Moreover, as shown in Table 1, the result from Harman’s one-factor test by using a CFA in AMOS 22 indicates that a one-factor model, in which all the items of four focal variables loaded onto the same factor, did not fit the data well (χ^2^/*df* = 18.20; CFI = 0.36; TLI = 0.32; SRMR = 0.28; RMSEA = 0.21). We also used a latent marker variable to examine the extent to which common method bias existed in the present study following Williams et al. (2010) procedures. The results suggested that the model with a single unmeasured latent method factor has no significant difference from the measurement model. Thus, these statistical results show that common method bias did not affect the findings of the present study.

### Descriptive Statistics

Means, standard deviations, and bivariate correlations for all study variables are presented in Table 2. The correlation coefficients among the predictors do not exceed 0.60, suggesting that the multicollinearity among the research variables is probably not severe (Nunnally, 1978). Cronbach’s alphas in this study ranged from 0.91 to 0.96.

**TABLE 2:** Descriptive statistics and correlations among variables used in the analyses.

**Variables**	***M***	***SD***	**1**	**2**	**3**	**4**	**5**	**6**	**7**	**8**	**9**	**10**
1 Gender^a^	1.47	0.50	–									
2 Age^b^	28.61	6.20	–0.09	–								
3 Education level^c^	3.99	1.10	–0.02	–0.17^∗∗^	–							
4 Tenure^d^	3.27	3.84	−0.12^∗^	0.55^∗∗∗^	–0.22^∗∗∗^	–						
5 OC	4.29	1.28	0.04	–0.06	0.02	–0.06	(0.94)					
6 Environment value	6.07	0.78	0.02	0.05	–0.05	0.05	0.15^∗∗^	(0.93)				
7 EMP	5.08	0.99	–0.01	0.05	–0.04	0.16^∗∗^	0.04	0.21^∗∗∗^	(0.96)			
8 PO	3.88	1.41	0.02	0.09	–0.04	0.13^∗^	0.03	0.12^∗^	0.57^∗∗∗^	(0.91)		
9 FTP	5.52	0.83	0.01	0.05	–0.08	0.08	0.00	0.36^∗∗∗^	0.18^∗∗^	0.01	(0.92)	
10 OCBE	4.63	1.37	–0.02	0.07	–0.04	0.21^∗∗^	–0.02	0.23^∗∗∗^	0.29^∗∗∗^	0.31^∗∗∗^	0.39^∗∗∗^	(0.94)

**n* = 374; ^∗^*p* < 0.05, ^∗∗^*p* < 0.01, ^∗∗∗^*p* < 0.001. OC, organizational commitment; EMP, empowering leadership; PO, psychological ownership; FTP, future time perspective; OCBE, organizational citizenship behavior toward the environment. ^*a*^For gender, 1 = “male” and 2 = “female.” ^*b*^For age, 1 = “below 18,” 2 = “18–25,” 3 = “26–30,” 4 = “31–40,” 5 = “41–50,” 6 = “51–60,” and 7 = “above 60.” ^*c*^For education level, 1 = “high school and below,” 2 = “college degree,” 3 = “bachelor degree,” 4 = “master’s degree,” and 5 = “doctoral degree.” ^*d*^Organizational tenure measured in years.*

As expected, empowering leadership was positively related with psychological ownership (*r* = 0.57; *p* < 0.001) and OCBE (*r* = 0.29; *p* < 0.001); psychological ownership was positively associated with OCBE (*r* = 0.31; *p* < 0.001). The results provided preliminary support for Hypotheses 1 and 2.

### Hypothesis Tests

Table 3 presents the results of the hierarchical regression analysis on psychological ownership and OCBEs. As shown in model 2b, empowering leadership was positively related with OCBE (β = 0.23; *p* < 0.001), even after taking into account the control variables. Hypothesis 1 was therefore supported.

**TABLE 3:** Hierarchical regression analysis results.

**Variables**	**DV = PO**		**DV = OCBE**				
	**Model 1a**	**Model 1b**	**Model 2a**	**Model 2b**	**Model 2c**	**Model 2d**	**Model 2e**
	***B (SE)***	***B (SE)***	***B (SE)***	***B (SE)***	***B (SE)***	***B (SE)***	***B (SE)***
Gender	0.03 (0.15)	0.04 (0.11)	−0.04(0.14)	−0.04(0.14)	−0.05(0.13)	−0.04(0.13)	−0.03(0.12)
Age	0.07 (0.11)	0.04 (0.09)	0.19^∗∗^(0.12)	0.17^∗∗^(0.11)	0.15^∗^(0.11)	0.10 (0.10)	0.10 (0.10)
Education level	−0.01(0.07)	0.00 (0.06)	−0.06(0.07)	−0.05(0.11)	−0.05(0.06)	−0.02(0.06)	−0.01(0.06)
Tenure	0.04 (0.02)	0.05 (0.02)	−0.11(0.03)	−0.11(0.03)	−0.14(0.03)	−0.07(0.02)	−0.07(0.02)
OC	0.13^∗^(0.06)	0.04 (0.05)	0.20^∗⁣∗∗^(0.05)	0.16^∗∗^(0.05)	0.16^∗∗^(0.05)	0.14^∗∗^(0.05)	0.15^∗∗^(0.05)
Environment value	0.10^∗^(0.09)	−0.01(0.08)	0.22^∗⁣∗∗^(0.09)	0.18^∗⁣∗∗^(0.09)	0.18^∗⁣∗∗^(0.09)	0.07 (0.09)	0.11^∗^(0.09)
EMP		0.56^∗∗∗^ (0.06)		0.23^∗∗∗^ (0.07)	0.11 (0.08)	0.06 (0.08)	0.03 (0.08)
PO					0.21^∗∗∗^ (0.06)	0.25^∗⁣∗∗^(0.05)	0.30^∗⁣∗∗^(0.06)
FTP						0.33^∗⁣∗∗^(0.08)	0.27^∗⁣∗∗^(0.08)
PO × FTP							0.21^∗∗∗^ (0.06)
Adjusted *R*^2^	0.02	0.32	0.10	0.15	0.18	0.26	0.30
△*R*^2^	0.02	0.30^∗∗∗^	0.10^∗∗∗^	0.05^∗∗∗^	0.03^∗∗∗^	0.08^∗∗∗^	0.04^∗∗∗^
△*F*	1.39^∗^	159.40^∗∗^	7.81^∗∗∗^	20.77^∗∗∗^	13.46^∗∗∗^	43.97^∗∗∗^	19.77^∗∗∗^

**N* = 374; ^∗^*p* < 0.05, ^∗∗^*p* < 0.01, ^∗∗∗^*p* < 0.001. OC, organizational commitment; EMP, empowering leadership; PO, psychological ownership; FTP, future time perspective; OCBE, organizational citizenship behavior toward the environment.*

As shown in model 1b, empowering leadership was significantly associated with psychological ownership (β = 0.56; *p* < 0.001), even after considering control variables. In model 2c, when control variables, empowering leadership and psychological ownership, were entered into the regression model simultaneously, psychological ownership was positively related with OCBE (β = 0.21; *p* < 0.001), supporting the mediating role of psychological ownership in the relationship between empowering leadership and OCBE. In model 2c, when psychological ownership was entered into the regression model, the significant relationship between empowering leadership (β = 0.23; *p* < 0.001, in model 2b) becomes non-significant (β = 0.11; *p* > 0.05, in model 2c), supporting the mediating role of psychological ownership (Baron and Kenny, 1986). Further, to test Hypothesis 2 in an integrated fashion, we performed a bootstrapping procedure by using the PROCESS macro for SPSS (Hayes, 2013). We first entered gender, age, education level, organizational tenure, organizational commitment, and environment value as controls; empowering leadership as the predictor; employee psychological ownership as the mediator; and OCBE as the dependent variable in model 4 of the PROCESS macro. Then, we set the bootstrap sample to 5,000 and select the “Mean center for construction of products” in options, by which use variables can be automatically mean-centered prior to construction of products. The results showed that the bootstrapping indirect effect size is 0.17 and 95% bootstrapping confidence intervals were (0.07, 0.27), providing the evidence for Hypothesis 2.

**FIGURE 2:**
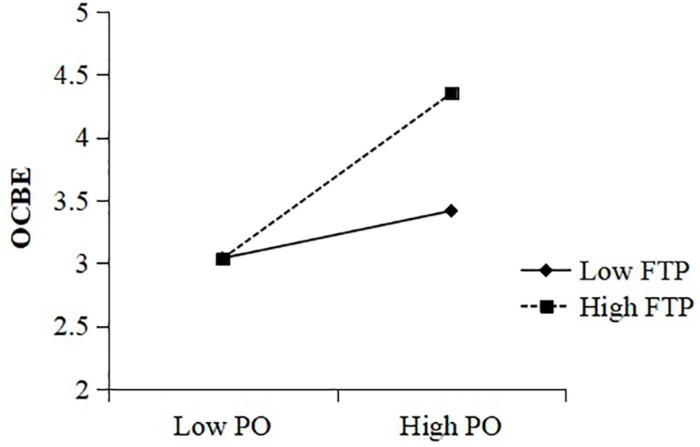
Interactive effects of PO and FTP on OCBE.

To test Hypothesis 3, which states that employee future time perspective is a second-stage moderator, we tested the regression of OCBE on a simple two-way interaction term (i.e., psychological ownership × future time perspective). As is evident from model 2e in Table 3, the interaction effect is positive and significant (β = 0.21; *p* < 0.001) and explained significantly more variance than the base model in which the interaction term was not included (Δ*R*^2^ = 0.04, *p* < 0.001), providing support for Hypothesis 3. To facilitate the interpretation of the moderation effect (Aiken et al., 1991), we plotted the relationship between psychological ownership and OCBE for low (-1 SD) and high (+1 SD) levels of future time perspective in Figure 2; the form of the interaction corroborated the predicted pattern, with the linkage between psychological ownership and OCBE being more pronounced for those with high rather than low future time perspective. Thus, Hypothesis 3 was supported.

To test Hypothesis 4 (i.e., the moderated mediation relationship) in an integrated fashion, we again used the PROCESS macro for SPSS (Hayes, 2013). We first entered gender, age, education level, organizational tenure, organizational commitment, and environment value as controls; empowering leadership as the predictor; employee psychological ownership as the mediator; employee future time perspective as the second stage moderator; and OCBE as the dependent variable in model 14 of the PROCESS macro. Then, we set the bootstrap sample to 5,000 and select the “Mean center for construction of products” in options, by which use variables can be automatically mean-centered prior to construction of products. Table 4 depicts the results of the conditional indirect relationship of empowering leadership and OCBE through employee psychological ownership at different values of future time perspective. As shown, when employees hold high (+ 1 SD) future time perspective, the indirect effect is significant [bootstrapping indirect effect = 0.34, *SE* = 0.06, 95% CI (0.22,0.45), excluding 0]. Although results also suggest that when employees hold low (-1 SD) future time perspective, the indirect effect was also significant [bootstrapping indirect effect = 0.15, *SE* = 0.06, 95% CI (0.03,0.26), excluding 0], the index of moderated mediation was significant [moderated mediation index = 0.17, *SE* = 0.06, 95% CI (0.06,0.31)], suggesting that the strength of two conditional indirect effects is significantly different (i.e., 0.34 vs. 0.15). These results provide support for Hypothesis 4. Table 5 shows the summary of the hypothesis test results.

**TABLE 4:** Conditional indirect effect of EMP on OCBE through PO at different values of FTP.

**FTP**	**EMP → PO → OCBE**			
	**Boot indirect effect**	**Boot SE**	**Boot LLCI**	**Boot ULCI**
−1 SD	0.15	0.06	0.03	0.26
Mean	0.26	0.05	0.16	0.35
+ 1 SD	0.34	0.06	0.22	0.45

*Controlling for gender, age, education level, position tenure, and dyad tenure. SE, standard error; −1 SD, one standard deviation below the mean value of FTP; Mean, mean value of FTP; +1 SD, one standard deviation above the mean value of FTP. Bootstrap *n* = 5,000. EMP, empowering leadership; PO, psychological ownership; FTP, future time perspective; OCBE, organizational citizenship behavior toward the environment; SE, standard error; LLCI, lower limit confidence interval; ULCI, upper limit confidence interval.*

**TABLE 5:** Summary of the hypotheses test results.

**Hypothesis**	**Estimate**	**Result**
H1: Empowering leadership is positively associated with citizenship behavior toward the environment (OCBE)	0.23^∗∗∗^	Supported
H2: Psychological ownership mediates the relationship between empowering leadership and OCBE	0.17^∗∗^	Supported
H3: Future time perspective moderates the relationships between psychological ownership and OCBE such that the relationship will be stronger for those high in future time perspective	0.21^∗∗∗^	Supported
H4: Employee future time perspective moderates the positive and indirect relationship between empowering leadership and employee OCBE through employee psychological ownership. Specifically, the indirect positive effect through employee psychological ownership will be stronger when employee has high rather than low future time perspectives	0.17^∗∗^	Supported

**N* = 374; ^∗∗^*p* < 0.01, ^∗∗∗^*p* < 0.001.*

## Discussion

To enhance employee OCBE is considered essential to the environmental performance and sustainability of organizations (Lamm et al., 2013; Lulfs and Hahn, 2013). We identified the role played by leaders in facilitating employee OCBEs from the leadership style approach. Specifically, the study investigated the influence of empowering leadership on followers’ OCBEs, drawing on the theory of psychological ownership, and addressed the moderating role of employee future time perspective.

The study found that empowering leadership is positively related to employee OCBEs (β = 0.23, *p* < 0.001). To detangle the effect of empowering leadership, we examined the mediating role played by psychological ownership and found a significant indirect effect of empowering leadership on OCBEs through psychological ownership [bootstrapping indirect effect = 0.17, 95% CI (0.07,0.27)]. Findings also supported the moderated mediation model; that is, the positive relationship between empowering leadership and OCBEs through psychological ownership is stronger when employees hold high future time perspective [bootstrapping indirect effect = 0.34, 95% CI (0.22,0.45)] than when they hold low future time perspective [bootstrapping indirect effect = 0.15, 95% CI (0.03,0.26)]; the moderated mediation effect is significant [*r* = 0.17, *SE* = 0.06, 95% CI (0.06,0.31)]. The study supported all the proposed hypotheses.

### Theoretical Implications

First, we found that empowering leadership is significantly related to employee OCBEs, which will enrich the environment management research by explaining the leader effect from the leadership style approach, since previous studies concerning leaders’ role in employee OCBEs have largely focused on how employee-perceived manager support affects employee OCBEs (Smith and O’Sullivan, 2012; Paillé and Boiral, 2013; Lamm et al., 2015; Saifulina and Carballo-Penela, 2017; Wesselink et al., 2017; Priyankara et al., 2018). This implies that employee proactive pro-environmental behaviors can be influenced by the leadership style of the managers.

Second, the present study draws on psychological ownership theory (Pierce et al., 2001, 2003; Van Dyne and Pierce, 2004) to clarify why empowering leadership will elevate employee OCBEs, offering a different theoretical lens to explain employee OCBEs, supplementing existing perspectives from social exchange theory (Paillé and Mejía-Morelos, 2014; Temminck et al., 2015; Priyankara et al., 2018), planned behavior theory (Greaves et al., 2013), and self-determination theory (Graves et al., 2013).

Third, the study shows that individual attitude (i.e., future time perspective) works as a synergistic effect interacting with psychological ownership on employee OCBEs. Individual difference such as environmental concern has been examined as a significant predictor of employee OCBEs (Daily et al., 2009; Temminck et al., 2015); here, we found that individual attitude interplaying with other situational factors revealed synergistic gains. Therefore, we linked empowering leadership, psychological ownership, individual attitude, and employee pro-environmental behavior literature that have developed separately and considered the individual–contextual interactionist factor for employee OCBEs.

Finally, the present study contributes to the empowering leadership literature by examining whether employees are more inclined to conduct discretionary green behaviors (i.e., OCBE) when empowered by leaders, suggesting the environmental implication of empowering leadership. Empowering leadership has been shown to associate with employee proactive behaviors directly beneficial to organizational effectiveness, such as knowledge exchange between employees (Srivastava et al., 2006), creativity (Zhang and Bartol, 2010), and in-role performance (Kim and Beehr, 2017). The present study linking empowering leadership and OCBEs demonstrates the pro-environmental potential of empowering at work.

### Managerial Implications

The present study also offers some practical implications for organizations that value environmental sustainability. First, our finding indicated that in the condition of leaders highlighting the meaningfulness of work, fostering participation in decision making, expressing confidence in high performance, and providing autonomy from bureaucratic constraints (Ahearne et al., 2005), employees are more likely to engage in OCBEs. Therefore, managers could consider ways to provide followers with autonomy and then increase employee OCBEs as a result. For example, managers might encourage employees to express their opinions when making decisions and might delegate more autonomy to followers at work.

Second, the study shows that when employees experience psychological ownership toward the organization, they are more attached to, protective of, and responsible for their organization (Van Dyne and Pierce, 2004) and engage in more OCBEs. Thus, managers should take steps to foster employees’ psychological ownership. According to psychological theory (Pierce et al., 2001, 2003), there are three main routes to give rise to employee psychological ownership: experiencing control over the organization, getting to know or becoming familiar with the organization, and investing the self into the organization. In management practice, our study found that empowering leadership can increase employee psychological ownership. Transactional leadership styles (Avey et al., 2009; Bernhard and O’Driscoll, 2011), ethical leadership (Avey et al., 2012), and benevolent leadership (Zhu et al., 2013) were also shown to promote psychological ownership. Moreover, managers can motivate employee psychological ownership by offering employee participation in decision making (Chi and Han, 2008; Han et al., 2010; Liu et al., 2012) or by providing employee profit-sharing schemes (Chi and Han, 2008) and stock ownership schemes (Chiu et al., 2007).

Third, the results show that employee future time perspective can strengthen the positive link between psychological ownership and employee OCBEs. So managers can highlight the future orientation in the workplace to direct employees’ attention to future goals or select employees who are high in future time perspective during recruitment.

### Limitations and Suggestions for Future Research

There are several limitations to be considered. The first limitation concerns the self-reported measurement of the focal variables in the study, which is not free from potentially having common method biases (Podsakoff et al., 2003), although self-rating of OCBE is widely used as it is a self-initial behavior that may not be perceived by others and we have taken some procedures to reduce common method bias (e.g., using a time-lag design, allowing respondent anonymity, and reducing evaluation apprehension) and performed statistical analyses to assess the severity of common method bias (Podsakoff et al., 2003; Williams et al., 2010). Future studies should obtain objective ratings or collect data from multiple sources or use an employee mixed-method approach. The second limitation involves the cross-sectional design and causal relationship in our study. Future studies can improve on it by using a longitudinal study design or finely designed experiment. Third, the present study only considered the influence of empowering leadership on employee OCBEs; future studies can make contributions by investigating the effect of other leadership styles, such as leader humility (Owens and Hekman, 2012), or negative leadership practice (e.g., abusive supervision). Moreover, the present study found that individual future time perspective can strengthen the positive relationship between factors and employee OCBEs; future research can examine another individual attitude that may affect employees’ pro-environmental engagement directly or by interacting with other contextual factors.

## Conclusion

Present organizations are confronted with increasing social responsibility to contribute to environmental sustainability. Employee OCBEs are considered essential to organizational environment performance. In the present investigation, we examined the positive relationship between empowering leadership and OCBEs through the mechanism of employee psychological ownership. Further, we found that the indirect effect of empowering leadership on OCBEs through psychological ownership is stronger when employees hold high rather than low future time perspectives. Thus, our study suggests why and when employee discretionary pro-environment behaviors in the workplace are influenced by empowering leadership, which may be overlooked in previous studies, and provides some insightful theoretical and practical implications.

## Data Availability Statement

The datasets generated for this study are available on request to the corresponding author.

## Ethics Statement

The studies involving human participants were reviewed and approved by the Ethics Committee of Wuhan University of Technology. Written informed consent was inferred through the participation of the online study.

## Author Contributions

MJ contributed to the conception, data curation, formal analysis, and writing and editing of the manuscript. HW contributed to the revision of the manuscript. ML contributed to the conception, and original draft and revision of the manuscript.

## Conflict of Interest

The authors declare that the research was conducted in the absence of any commercial or financial relationships that could be construed as a potential conflict of interest.

## References

[B1] Aguinis H. Glavas A. (2012). What we know and don’t know about corporate social responsibility. *J. Manag.* 38 932–968. 10.1177/0149206311436079

[B2] Ahearne M. Mathieu J. Rapp A. (2005). To empower or not to empower your sales force? An empirical examination of the influence of leadership empowerment behavior on customer satisfaction and performance. *J. Appl. Psychol.* 90 945–955. 10.1037/0021-9010.90.5.945 16162066

[B3] Aiken L. S. West S. G. Reno R. R. (1991). *Multiple Regression: Testing and Interpreting Interactions.* Thousand Oaks, CA: Sage.

[B4] Allen N. J. Meyer J. P. (1990). The measurement and antecedents of affective, continuance and normative commitment to the organization. *J. Occup. Psychol.* 63 1–18.

[B5] Avey J. B. Avolio B. J. Crossley C. D. Luthans F. (2009). Psychological ownership: theoretical extensions, measurement and relation to work outcomes. *J. Organ. Behav.* 30 173–191. 10.1002/job.583

[B6] Avey J. B. Wernsing T. S. Palanski M. E. (2012). Exploring the process of ethical leadership: the mediating role of employee voice and psychological ownership. *J. Bus. Ethics* 107 21–34. 10.1007/s10551-012-1298-2

[B7] Baron R. M. Kenny D. A. (1986). The moderator–mediator variable distinction in social psychological research: conceptual, strategic, and statistical considerations. *J. Pers. Soc. Psychol.* 51 1173–1182. 3806354 10.1037//0022-3514.51.6.1173

[B8] Bernhard F. O’Driscoll M. P. (2011). Psychological ownership in small family-owned businesses: leadership style and nonfamily-employees’ work attitudes and behaviors. *Group Organ. Manag.* 36 345–384. 10.1177/1059601111402684

[B9] Bissing-Olson M. J. Iyer A. Fielding K. S. Zacher H. (2013). Relationships between daily affect and pro-environmental behavior at work: the moderating role of pro-environmental attitude. *J. Organ. Behav.* 34 156–175. 10.1002/job.1788

[B10] Blau P. M. (1964). *Exchange and Power in Social Life.* New York, NY: Wiley.

[B11] Boiral O. (2009). Greening the corporation through organizational citizenship behaviors. *J. Bus. Ethics* 87 221–236. 10.1007/s10551-008-9881-2

[B12] Boiral O. Paille P. (2012). Organizational citizenship behaviour for the environment: measurement and validation. *J. Bus. Ethics* 109 431–445. 10.1007/s10551-011-1138-9

[B13] Brislin R. W. (1980). “Translation and content analysis of oral and written material,” in *Handbook of Crosscultural Psychology: Methodology*, eds Triandis H. C. Berry J. W., (Boston, MA: Allyn and Bacon), 349–444.

[B14] Chi N.-W. Han T.-S. (2008). Exploring the linkages between formal ownership and psychological ownership for the organization: the mediating role of organizational justice. *J. Occup. Organ. Psychol.* 81 691–711. 10.1348/096317907x262314

[B15] Chiu W. C. K. Harry Hui C. Lai G. W. F. (2007). Psychological ownership and organizational optimism amid China’s corporate transformation: effects of an employee ownership scheme and a management-dominated board. *Int. J. Hum. Resour. Manag.* 18 303–320. 10.1080/09585190601102539

[B16] Cropanzano R. Mitchell M. S. (2005). Social exchange theory: an interdisciplinary review. *J. Manag.* 31 874–900. 10.1177/0149206305279602

[B17] Daily B. F. Bishop J. W. Govindarajulu N. (2009). A conceptual model for organizational citizenship behavior directed toward the environment. *Bus. Soc.* 48 243–256. 10.1177/0007650308315439

[B18] de Volder M. L. Lens W. (1982). Academic achievement and future time perspective as a cognitive-motivational concept. *J. Pers. Soc. Psychol.* 42 566–571. 10.1037/0022-3514.42.3.566

[B19] Dilchert S. Ones D. S. (2012). Environmental sustainability in and of organizations. *Indust. Organ. Psychol. Perspect. Sci. Pract.* 5 503–511.

[B20] Edwards J. R. Lambert L. S. (2007). Methods for integrating moderation and mediation: a general analytical framework using moderated path analysis. *Psychol. Methods* 12 1–22. 10.1037/1082-989x.12.1.1 17402809

[B21] Erdogan B. Bauer T. N. Taylor S. (2015). Management commitment to the ecological environment and employees: implications for employee attitudes and citizenship behaviors. *Hum. Relat.* 68 1669–1691. 10.5465/ambpp.2013.16538abstract

[B22] Fukukawa K. Shafer W. E. Lee G. M. (2007). Values and attitudes toward social and environmental accountability: a study of MBA students. *J. Bus. Ethics* 71 381–394. 10.1007/s10551-005-3893-y

[B23] Galpin T. Lee Whittington J. (2012). Sustainability leadership: from strategy to results. *J. Bus. Strategy* 33 40–48. 10.1108/02756661211242690

[B24] Gao A. Jiang J. (2019). Perceived empowering leadership, harmonious passion, and employee voice: the moderating role of job autonomy. *Front. Psychol.* 10:1484. 10.3389/fpsyg.2019.01484 31379639 PMC6660284

[B25] Giorgi G. Arcangeli G. Mucci N. Cupelli V. (2015). Economic stress in the workplace: the impact of fear of the crisis on mental health. *Work* 51 135–142. 10.3233/WOR-141844 24594539

[B26] Gjesme T. (1979). Future time orientation as a function of achievement motives, ability, delay of gratification, and sex. *J. Psychol.* 101 173–188.

[B27] Graves L. M. Sarkis J. Zhu Q. (2013). How transformational leadership and employee motivation combine to predict employee proenvironmental behaviors in China. *J. Environ. Psychol.* 35 81–91. 10.1016/j.jenvp.2013.05.002

[B28] Greaves M. Zibarras L. D. Stride C. (2013). Using the theory of planned behavior to explore environmental behavioral intentions in the workplace. *J. Environ. Psychol.* 34 109–120. 10.1016/j.jenvp.2013.02.003

[B29] Griffin M. A. Neal A. Parker S. K. (2007). A new model of work role performance: positive behavior in uncertain and interdependent contexts. *Acad. Manag. J.* 50 327–347.

[B30] Han T.-S. Chiang H.-H. Chang A. (2010). Employee participation in decision making, psychological ownership and knowledge sharing: mediating role of organizational commitment in Taiwanese high-tech organizations. *Int. J. Hum. Resour. Manag.* 21 2218–2233. 10.1080/09585192.2010.509625

[B31] Haugh H. M. Talwar A. (2010). How do corporations embed sustainability across the organization? *Acad. Manag. Learn. Educ.* 9 384–396. 10.5465/Amle.2010.53791822

[B32] Hayes A. F. (2013). *Introduction to Mediation, Moderation, and Conditional Process Analysis: a Regression-Based Approach.* New York, NY: Guilford Press.

[B33] Hofstede G. (2003). What is culture? a reply to Baskerville. *Account. Organ. Soc.* 28 811–813. 10.1016/s0361-3682(03)00018-7

[B34] Husman J. Lens W. (1999). The role of the future in student motivation. *Educ. Psychol.* 34 113–125. 10.1207/s15326985ep3402_4

[B35] Jenkin T. A. McShane L. Webster J. (2011a). Green information technologies and systems: employees’ perceptions of organizational practices. *Bus. Soc.* 50 266–314. 10.1038/s41586-019-1545-0 31619795 PMC6800389

[B36] Jenkin T. A. Webster J. McShane L. (2011b). An agenda for ‘Green’information technology and systems research. *Inform. Organ.* 21 17–40.

[B37] Kim A. Kim Y. Han K. Jackson S. E. Ployhart R. E. (2017). Multilevel influences on voluntary workplace green behavior: individual differences, leader behavior, and coworker advocacy. *J. Manag.* 43 1335–1358. 10.1177/0149206314547386

[B38] Kim M. Beehr T. A. (2017). Self-efficacy and psychological ownership mediate the effects of empowering leadership on both good and bad employee behaviors. *J. Leadersh. Organ. Stud.* 24 466–478. 10.1177/1548051817702078

[B39] Kooij D. Kanfer R. Betts M. Rudolph C. W. (2018). Future time perspective: a systematic review and meta-analysis. *J. Appl. Psychol.* 103 867–893. 10.1037/apl0000306 29683685

[B40] Lamm E. Tosti-Kharas J. King C. E. (2015). Empowering employee sustainability: perceived organizational support toward the environment. *J. Bus. Ethics* 128 207–220. 10.1007/s10551-014-2093-z

[B41] Lamm E. Tosti-Kharas J. Williams E. G. (2013). Read this article, but don’t print it. *Group Organ. Manag.* 38 163–197. 10.1177/1059601112475210

[B42] Leach D. J. Wall T. D. Jackson P. R. (2003). The effect of empowerment on job knowledge: an empirical test involving operators of complex technology. *J. Occup. Organ. Psychol.* 76 27–52.

[B43] LePine J. A. Erez A. Johnson D. E. (2002). The nature and dimensionality of organizational citizenship behavior: a critical review and meta-analysis. *J. Appl. Psychol.* 87 52–65. 10.1037/0021-9010.87.1.52 11916216

[B44] LePine M. A. Zhang Y. W. Crawford E. R. Rich B. L. (2016). Turning their pain to gain: charismatic leader influence on follower stress appraisal and job performance. *Acad. Manag. J.* 59 1036–1059.

[B45] Liu J. Wang H. Hui C. Lee C. (2012). Psychological ownership: how having control matters. *J. Manag. Stud.* 49 869–895. 10.1111/j.1467-6486.2011.01028.x 25983713

[B46] Lulfs R. Hahn R. (2013). Corporate greening beyond formal programs, initiatives, and systems: a conceptual model for voluntary pro-environmental behavior of employees. *Eur. Manag. Rev.* 10 83–98. 10.1111/emre.12008

[B47] Maignan I. Ferrell O. C. Hult G. T. M. (1999). Corporate citizenship: cultural antecedents and business benefits. *J. Acad. Mark. Sci.* 27 455–469.

[B48] Martin S. L. Liao H. Campbell E. M. (2013). Directive versus empowering leadership: a field experiment comparing impacts on task proficiency and proactivity. *Acad. Manag. J.* 56 1372–1395. 10.5465/amj.2011.0113

[B49] Mayhew M. G. Ashkanasy N. M. Bramble T. Gardner J. (2007). A study of the antecedents and consequences of psychological ownership in organizational settings. *J. Soc. Psychol.* 147 477–500. 10.3200/SOCP.147.5.477-500 18225830

[B50] McColl-Kennedy J. R. Anderson R. D. (2002). Impact of leadership style and emotions on subordinate performance. *Leadersh. Q.* 13 545–559.

[B51] McCormick L. Donohue R. (2019). Antecedents of affective and normative commitment of organisational volunteers. *Int. J. Hum. Resour. Manag.* 30 2581–2604. 10.1080/09585192.2016.1166388

[B52] Mesmer-Magnus J. Viwsevaran C. Wiernik B. M. (2012). “The role of commitment in bridging the gap between organizational sustainability and environmental sustainability,” in *Managing Human Resources for Environmental Sustainability*, eds Jackson S. E. Ones D. S. Dilchert S., (San Francisco, CA: Jossey-Bass).

[B53] Mischel W. (1961). Delay of gratification, need for achievement, and acquiescence in another culture. *J. Abnorm. Soc. Psychol.* 62 543–552. 14474527 10.1037/h0039842

[B54] Mucci N. Giorgi G. Roncaioli M. Fiz Perez J. Arcangeli G. (2016). The correlation between stress and economic crisis: a systematic review. *Neuropsychiatr. Dis. Treat* 12 983–993. 10.2147/NDT.S98525 27143898 PMC4844458

[B55] Norton T. A. Parker S. L. Zacher H. Ashkanasy N. M. (2015). Employee green behavior. *Organ. Environ.* 28 103–125. 10.1177/1086026615575773

[B56] Nunnally J. C. (1978). *Psychometric Theory.* New York, NY: MacGrow-Hill.

[B57] O’driscoll M. P. Pierce J. L. Coghlan A.-M. (2006). The psychology of ownership: work environment structure, organizational commitment, and citizenship behaviors. *Group Organ. Manag.* 31 388–416. 10.1177/1059601104273066

[B58] Organ D. W. (1998). *Organizational Citizenship Behavior: The Good Soldier Syndrome.* Lexington, MA: Lexington Books.

[B59] Owens B. P. Hekman D. R. (2012). Modeling how to grow: an inductive examination of humble leader behaviors, contingencies, and outcomes. *Acad. Manag. J.* 55 787–818. 10.5465/amj.2010.0441

[B60] Paillé P. Boiral O. (2013). Pro-environmental behavior at work: construct validity and determinants. *J. Environ Psychol.* 36 118–128. 10.1016/j.jenvp.2013.07.014

[B61] Paille P. Boiral O. Chen Y. (2013). Linking environmental management practices and organizational citizenship behaviour for the environment: a social exchange perspective. *Int. J. Hum. Resour. Manag.* 24 3552–3575. 10.1080/09585192.2013.777934

[B62] Paillé P. Chen Y. Boiral O. Jin J. (2013). The impact of human resource management on environmental performance: an employee-level study. *J. Bus. Ethics* 121 451–466. 10.1007/s10551-013-1732-0

[B63] Paillé P. Mejía-Morelos J. H. (2014). Antecedents of pro-environmental behaviours at work: the moderating influence of psychological contract breach. *J. Environ. Psychol.* 38 124–131. 10.1016/j.jenvp.2014.01.004

[B64] Peng H. Pierce J. (2015). Job- and organization-based psychological ownership: relationship and outcomes. *J. Manag. Psychol.* 30 151–168. 10.1108/jmp-07-2012-0201 18225830

[B65] Pierce J. L. Kostova T. Dirks K. T. (2001). Toward a theory of psychological ownership in organizations. *Acad. Manag. Rev.* 26 298–310. 10.5465/Amr.2001.4378028 26052784

[B66] Pierce J. L. Kostova T. Dirks K. T. (2003). The state of psychological ownership: integrating and extending a century of research. *Rev. Gen. Psychol.* 7 84–107. 10.1037/1089-2680.7.1.84

[B67] Podsakoff P. M. MacKenzie S. B. Lee J.-Y. Podsakoff N. P. (2003). Common method biases in behavioral research: a critical review of the literature and recommended remedies. *J. Appl. Psychol.* 88 879–903. 10.1037/0021-9010.88.5.879 14516251

[B68] Podsakoff P. M. MacKenzie S. B. Podsakoff N. P. (2012). Sources of method bias in social science research and recommendations on how to control it. *Annu. Rev. Psychol.* 63 539–569. 10.1146/annurev-psych-120710-100452 21838546

[B69] Preacher K. J. Rucker D. D. Hayes A. F. (2007). Addressing moderated mediation hypotheses: theory, methods, and prescriptions. *Multivar. Behav. Res.* 42 185–227. 10.1080/00273170701341316 26821081

[B70] Priyankara H. Luo F. Saeed A. Nubuor S. Jayasuriya M. (2018). How does leader’s support for environment promote organizational citizenship behaviour for environment? a multi-theory perspective. *Sustainability* 10:271 10.3390/su10010271

[B71] Qian J. Lin X. Han Z. R. Tian B. Chen G. Z. Wang H. (2015). The impact of future time orientation on employees’ feedback-seeking behavior from supervisors and co-workers: the mediating role of psychological ownership. *J. Manag. Organ.* 21 336–349. 10.1017/jmo.2014.78

[B72] Qian J. Song B. Jin Z. Wang B. Chen H. (2018). Linking empowering leadership to task performance, taking charge, and voice: the mediating role of feedback-seeking. *Front. Psychol.* 9:2025. 10.3389/fpsyg.2018.02025 30410461 PMC6209672

[B73] Robert C. Probst T. M. Martocchio J. J. Drasgow F. Lawler J. J. (2000). Empowerment and continuous improvement in the United States, Mexico, Poland, and India: predicting fit on the basis of the dimensions of power distance and individualism. *J. Appl. Psychol.* 85 643–658. 10.1037/0021-9010.85.5.643 11055141

[B74] Saifulina N. Carballo-Penela A. (2017). Promoting sustainable development at an organizational level: an analysis of the drivers of workplace environmentally friendly behaviour of employees. *Sustain. Dev.* 25 299–310. 10.1002/sd.1654

[B75] Siemsen E. Roth A. Oliveira P. (2010). Common method bias in regression models with linear, quadratic, and interaction effects. *Organ. Res. Methods* 13 456–476. 10.1177/1094428109351241

[B76] Smith A. M. O’Sullivan T. (2012). Environmentally responsible behaviour in the workplace: an internal social marketing approach. *J. Mark. Manag.* 28 469–493. 10.1080/0267257x.2012.658837

[B77] Srivastava A. Bartol K. M. Locke E. A. (2006). Empowering leadership in management teams: effects on knowledge sharing, efficacy, and performance. *Acad. Manag. J.* 49 1239–1251. 10.5465/Amj.2006.23478718

[B78] Starik M. Marcus A. A. (2000). Introduction to the special research forum on the management of organizations in the natural environment: a field emerging from multiple paths, with many challenges ahead. *Acad. Manag. J.* 43 539–546.

[B79] Strathman A. Gleicher F. Boninger D. S. Edwards C. S. (1994). The consideration of future consequences: weighing immediate and distant outcomes of behavior. *J. Pers. Soc. Psychol.* 66 742–752. 10.1037/0022-3514.66.4.742

[B80] Stritch J. M. Christensen R. K. (2016). Going green in public organizations. *Am. Rev. Publ. Admin.* 46 337–355. 10.1177/0275074014552470

[B81] Takeuchi R. Tesluk P. E. Yun S. H. Lepak D. P. (2005). An integrative view of international experience. *Acad. Manag. J.* 48 85–100.

[B82] Tan J. X. Cham T. H. Zawawi D. Aziz Y. A. (2019). Antecedents of organizational citizenship behavior and the mediating effect of organization commitment in the hotel industry. *Asian J. Bus. Res.* 9 121–139. 10.14707/ajbr.190064

[B83] Temminck E. Mearns K. Fruhen L. (2015). Motivating employees towards sustainable behaviour. *Bus. Strateg. Environ.* 24 402–412. 10.1002/bse.1827

[B84] Tuan L. T. (2017). Activating tourists’ citizenship behavior for the environment: the roles of CSR and frontline employees’ citizenship behavior for the environment. *J. Sustain. Tourism* 26 1178–1203. 10.1080/09669582.2017.1330337

[B85] Turner J. R. Müller R. (2005). The project manager’s leadership style as a success factor on projects: a literature review. *Project Manag. J.* 36 49–61.

[B86] Van Dyne L. Pierce J. L. (2004). Psychological ownership and feelings of possession: three field studies predicting employee attitudes and organizational citizenship behavior. *J. Organ. Behav.* 25 439–459. 10.1002/job.249

[B87] Wang L. Law K. S. Zhang M. J. Li Y. N. Liang Y. (2019). It’s mine! Psychological ownership of one’s job explains positive and negative workplace outcomes of job engagement. *J. Appl. Psychol.* 104 229–246. 10.1037/apl0000337 30211569

[B88] Wesselink R. Blok V. Ringersma J. (2017). Pro-environmental behaviour in the workplace and the role of managers and organisation. *J. Cleaner Production* 168 1679–1687. 10.1016/j.jclepro.2017.08.214

[B89] Williams L. J. Hartman N. Cavazotte F. (2010). Method variance and marker variables: a review and comprehensive CFA marker technique. *Organ. Res. Methods* 13 477–514. 10.1177/1094428110366036

[B90] Zhang X. M. Bartol K. M. (2010). Linking empowering leadership and employee creativity: the influence of psychological empowerment, intrinsic motivation, and creative process engagement. *Acad. Manag. J.* 53 107–128. 10.5465/Amj.2010.48037118

[B91] Zhu H. Chen C. C. Li X. Zhou Y. (2013). From personal relationship to psychological ownership: the importance of manager–owner relationship closeness in family businesses. *Manag. Organ. Rev.* 9 295–318. 10.1111/more.12001

[B92] Zimbardo P. G. Boyd J. N. (1999). Putting time in perspective: a valid, reliable individual-differences metric. *J. Pers. Soc. Psychol.* 77 1271–1288.

